# Studies on Early Allergic Sensitization in the Lithuanian Birth Cohort

**DOI:** 10.1100/2012/909524

**Published:** 2012-04-19

**Authors:** Ruta Dubakiene, Odilija Rudzeviciene, Indre Butiene, Indre Sezaite, Malvina Petronyte, Dalia Vaicekauskaite, Aurelija Zvirbliene

**Affiliations:** ^1^Vilnius University Faculty of Medicine, University Street 3 01513, Vilnius, Lithuania; ^2^Institute of Biotechnology of Vilnius University, Graiciuno Street 8 02241, Vilnius, Lithuania

## Abstract

Cohort studies are of great importance in defining the mechanism responsible for the development of allergy-associated diseases, such as atopic dermatitis, allergic asthma, and allergic rhinoconjunctivitis. Although these disorders share genetic and environmental risk factors, it is still under debate whether they are linked or develop sequentially along an atopic pathway. The current study was aimed to determine the pattern of allergy sensitization in the Lithuanian birth cohort “Alergemol” (*n* = 1558) established as a part of the multicenter European birth cohort “EuroPrevall”. Early sensitization to food allergens in the “Alergemol” birth cohort was analysed. The analysis revealed 1.3% and 2.8% of symptomatic-sensitized subjects at 6 and 12 months of age, respectively. The sensitization pattern in response to different allergens in the group of infants with food allergy symptoms was studied using allergological methods *in vivo* and *in vitro*. The impact of maternal and environmental risk factors on the early development of food allergy in at 6 and 12 months of age was evaluated. Our data showed that maternal diet, diseases, the use of antibiotics, and tobacco smoke during pregnancy had no significant impact on the early sensitization to food allergens. However, infants of atopic mothers were significantly more often sensitized to egg as compared to the infants of nonatopic mothers.

## 1. Introduction

The allergic diseases are epidemically spread worldwide. The most common in childhood are asthma, allergic rhinoconjunctivitis, atopic dermatitis, and food allergy. The true prevalence and risk factors of food allergies in children are not fully understood. Nowadays food allergies are recognized as one of the most important causes of allergic diseases. The frequency of food allergies has increased significantly over the last twenty years and now affects almost 4% of the general population, irrespective of age [1]. Food allergies are most prevalent in young children, affecting about 6–8% of children younger than three years [2]. Although any food may trigger an allergic response, relatively few foods are responsible for most food allergies. In children, cow's milk, egg, peanut, soy, wheat, and fish cause more than 85% of confirmed food hypersensitivity reactions [3–8]. About 2.5% of infants are allergic to cow's milk, about 1.5% of young children to eggs and 0.5% to peanuts [3]. A family history of atopic diseases increases the risk of food allergy [9, 10]. Some food allergies, such as those to cow's milk and hen's egg, are usually outgrown during childhood, whereas peanut and tree nut allergies are more likely to persist into adulthood.

Cohort studies are of fundamental importance and value in defining the mechanism responsible for the development of allergy-associated diseases, such as atopic dermatitis, allergic asthma and allergic rhinoconjunctivitis [6, 11–15]. In particular birth cohort studies allow disease development to be studied through a longitudinal approach. Although these disorders share genetic and environmental risk factors, it is still under debate whether they are actually unrelated, develop sequentially along an atopic pathway, or are linked. It is known that the majority of children with atopic dermatitis or food allergy will lose their disease by school age. However, a certain proportion will develop allergic asthma and/or allergic rhinoconjunctivitis later in childhood. The mechanism of this atopic march is not well understood. Currently, the sensitization to hen's egg appears to be one of the best prognostic markers for later development of asthma and allergic rhinoconjunctivitis [14, 15]. Interestingly, early episodic wheezing by itself seems not to be associated with later asthma development, but the combination with early sensitization, especially to hen's egg, is a strong risk factor [3, 13, 16].

The current study was aimed to determine the pattern of allergy sensitization in the Lithuanian birth cohort “Alergemol” (*n* = 1558) established as a part of the multicenter “EuroPrevall” cohort that recruited a total of over 12000 newborns in 9 European countries. In the current study, we have analysed the prevalence of food allergies in infants at 6 and 12 months of age and statistically evaluated the relationship between maternal diet and diseases during pregnancy, the influence of environmental factors, parental allergy, and early sensitization to food allergens. 

## 2. Methods

The Lithuanian birth cohort “Alergemol” was established as a part of the multicenter “EuroPrevall” birth cohort that recruited over 12000 newborns in 9 European countries. The “EuroPreval” cohort was established in 2005–2009 using a standardised approach across 9 European countries, namely, the Iceland, UK, The Netherlands, Germany, Poland, Lithuania, Spain, Italy, and Greece. The recruitment of the Lithuanian birth cohort was performed in the Obstetrics and Gynecology Clinics of Vilnius City University Hospital in a period of January, 2006 and April, 2007. Total number of the recruited newborns in the Lithuanian birth cohort “Alergemol” was 1558.

Data have been collected on the maternal factors antenatally and at months 6 and 12. The questionnaires included data on preexisting diseases, intake of foods, nutritional supplements, medications, tobacco, sociodemographic data, pet ownership, and family history. In addition to three telephone interviews during the first months, parents were asked to immediately inform the allergology center about possible allergic reactions to food at any time during the followup period.

The study group included symptomatic infants sensitized to food allergens and their age-matched controls. Information was collected using parental questionnaires filled at the day of the recruitment, 12 months questionnaires and physical examination form, results of performed skin prick tests (SPT) and specific IgE (sIgE) analysis. All infants with suspected food allergy were clinically evaluated including double-blind placebo-controlled food challenge (DBPCFC) tests.

Skin prick tests were performed with commercial food allergens (ALK, Denmark) using standard methods [17].

Food-specific IgE were determined by using the ImmunoCAP with commercial diagnostic kits (Phadia, Sweden) according to the manufacturer‘s recommendations.

Statistical analysis of the collected data was performed using SAS 9.2 computer program.

The study was approved by the Vilnius Regional Committee of Biomedical Research (Lithuania, permission no. 6B-10–248.

## 3. Results and Discussion

The current study was aimed to investigate the pattern of food sensitization in the Lithuanian birth cohort “Alergemol” that represents a part of the multicenter “EuroPrevall” birth cohort. The “EuroPrevall” cohort has been specifically designed to examine food allergies in the first years of life. The Lithuanian birth cohort “Alergemol” (*n* = 1558) was established in 2006-2007. It follows up well-characterised children and their family members. To perform the complete clinical followup until the age of 12 months, data have been collected on maternal factors antenatally and at 6 and 12 months of age, such as preexisting diseases, intake of foods, nutritional supplements, medications, tobacco, sociodemographic data, pet ownership, and family history. As the main focus of the cohort study is the development of food allergies, data have been collected on breastfeeding, weaning and food intake of the infant, nutritional supplements, infections, the usage of medications, vaccinations, exposure to cigarette smoke, mould, and pets, and any signs and symptoms of allergic diseases. Extensive nutritional intake data have been collected regarding the length of time of breastfeeding and the complementary feeding overlap, timing of weaning, the order of allergen introduction, allergen frequency and dose, diversity of diet, brand of formula used, and commercial versus home-made meals. In addition, parents were requested to report any allergy-associated signs or symptoms, such as atopic dermatitis, gastrointestinal symptoms without fever, and wheezing.

In total, there were 320 phone calls to the allergology center about possible allergic reactions to food. One-hundred fifty four infants have been invited for clinical assessment. Forty-three patients with possible allergy symptoms attended the allergology center for a physical examination including the scoring of atopic dermatitis (SCORAD), completion of a standardised questionnaire, performance of skin prick tests and to give a blood sample for specific IgE analysis. Blood serum specimens were tested for common food-specific IgE.

Two age-matched control children were recruited from the pool of asymptomatic children and followed with similar evaluation excluding DBPCFC. In total, 85 age-matched children were invited for clinical investigation as controls. The characteristics of the Lithuanian birth cohort “Alergemol” at the day of the recruitment are presented in Table 1. The number of self-reported cases of food hypersensitivity of the family members ranged from 1% (fathers) to 9.7% (other family members). The rate of the Caesarean section was 15.7% in the cohort. Both parents were of similar age (mean age 28–30 years).

In the previous Lithuanian birth cohort PLANK-K that was recruited in 2004-2005 and analysed the socioeconomic and environmental risk factors for allergy development it was determined that the significant risk to develop allergy at early age depends on father's allergy, soft furniture in the environment, pollinosis in the family, and allergic disease in the family [9]. Previous cohort studies demonstrated that Lithuanian mothers of children suffering from allergy have a food allergy more often as compared to the group of healthy mothers (61,54% and 14,05% cases, resp., *P* < 0.001) [9]. Similar data on the impact of mother's food allergy on the development of allergy in children are reported in the other European birth cohorts: PIAMA (18), MASS-90 (6), KOALA (16), GINI (7), and LISA (20).

To evaluate the possible influence of environmental factors on the development of food allergy, the exposure of mothers and infants to cigarette smoke and pets has been analysed. The data are summarized in Table 2.

As presented in Table 2, the main population of the Lithuanian birth cohort is urban. About 10% of pregnant women included into the cohort were smoking or were exposed to a passive smoking. However, there was no significant influence on the tobacco smoke during pregnancy on early sensitization to food allergens (*P* = 0.25). More than 20% cohort participants had pets (cats and dogs) at home. Wood laminate in the dwellings was more common than a carpet (62.1% and 30.2%, resp.). The environmental risk factors for allergy sensitization in Lithuania were studied also in the previous PLANK-K cohort [9]. In the previous cohort the environmental factors in atopic/allergic groups as compared to the control (healthy) group were evaluated using the regression analysis. The family risk factors such as smoking (*P* = 0.1209/0.8927) and the** c**ontact with pets and animals (*P* = 0.0912/0.0845) were not significant for allergy sensitization. There were no significant differences between the impact of smoking on the allergy sensitization in children in healthy and allergic/atopic women (*P* = 0.1209/0.8927) [9]. Similar data were presented in the other cohorts, where children were exposed to tobacco smoke *in utero*. The rate of the exposure to tobacco smoke *in utero* varied from 8% to 12% in different countries [7, 12, 14, 15, 18]. Thus, the impact of environmental exposures on the risk of developing food allergy was not evidenced by the current study and by previous cohort studies.

**Figure 1:**
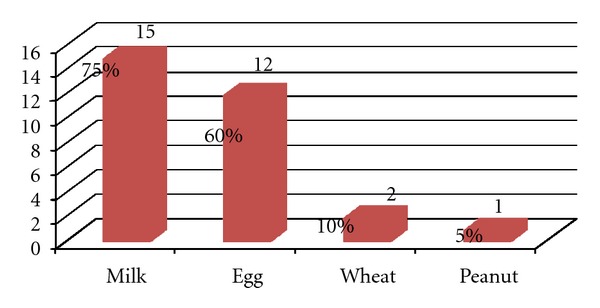
Sensitization to food allergens in the group of symptomatic infants at 6 months of age (*n* = 20).

Early sensitization to food allergy at 6 months of age in the “Alergemol” birth cohort has been analysed. The data are presented in the Figure 1.

**Figure 2:**
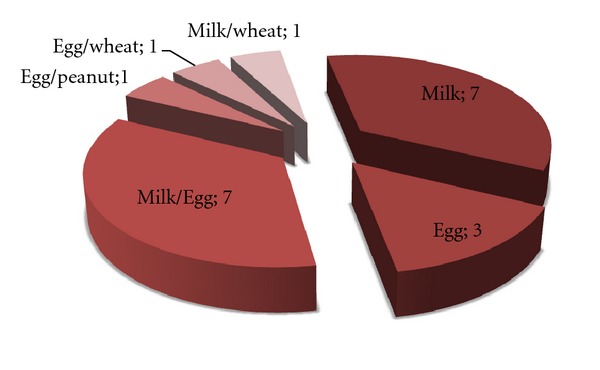
Sensitization to food allergens and their combinations (absolute numbers of sensitized infants) in the group of symptomatic infants at 6 months of age (*n* = 20).

 Early sensitization to food allergens was detected in 20 infants at 6 months of age (1.3%, 20 out of 1558). From this group, 15 (75%) symptomatic subjects were sensitized to milk, 12 (60%) symptomatic subjects were sensitized to egg. Sensitization to wheat was confirmed in 2 patients and to peanut—in 1 patient (Figure 2). In the group of 15 patients sensitized to milk, positive SPT was found in 5, elevated sIgE in 4, only immediate or repetitive symptoms were reported in 8 patients. In 12 patients sensitized to egg, positive SPT was found in 9, elevated sIgE in 7, only immediate or repetitive symptoms were reported in 1 patient. Sensitization to wheat was confirmed in 2 patients by SPT and reported symptoms and to the peanut—in 1 subject by elevated sIgE. The food allergy was confirmed by positive DBPCFC in 4 infants including 2 infants sensitized for milk, 1—for egg, 1—for wheat. One-half of the symptomatic patients (10/20) were sensitized to more than one allergen. Elevated milk sIgE was detected only for boys (*P* = 0.09), elevated milk and egg sIgE were detected more often for boys than the girls; however, the statistical difference was not significant (*P* = 0.08). Sensitization to food allergens at the age of 6 months was studied previously only in 2 cohorts: ALLADIN and DARC [19, 20]. There was a significant variation in the reported data: in the ALLADIN cohort the sensitization to food allergens was determined in 7% of infants at the age of 6 months [19], whereas in the DARC cohort, the sensitization rate at the same age group was only 0.4% [20]. Our study shows that maternal avoidance of milk and egg products during pregnancy as well as the use in elevated amounts of the product was not related to early sensitization to milk and egg allergens (*P* = 0.38). Similar data were reported in the other European birth cohorts such as PIAMA (18), MASS-90 (6), KOALA (16), GINI (7), and LISA (20).

**Figure 3:**
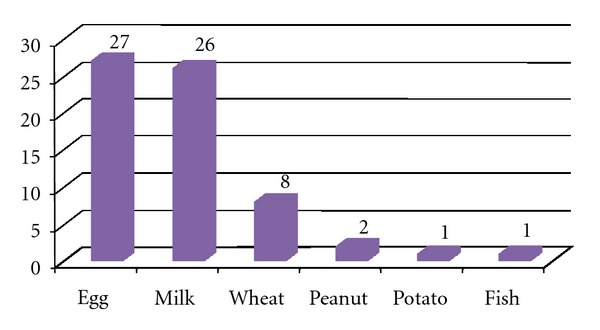
Sensitization to food allergens (absolute numbers of sensitized infants) in the group of symptomatic infants at 12 months of age (*n* = 43).

**Figure 4:**
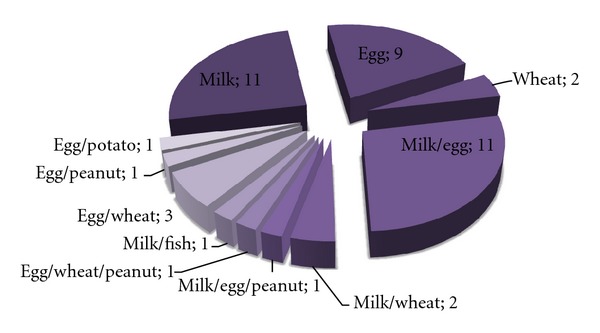
Sensitization to food allergens (absolute numbers of sensitized infants) in the group of symptomatic infants at 12 months of age (*n* = 43).

 The analysis of the food allergy sensitization pattern in children at 12 months of age revealed the increased number of symptomatic-sensitized subjects as compared to the group of children at 6 months of age (Figure 3). The sensitization to food allergens was detected in 43 children at 12 months of age (2.8%, 43 out of 1558) (Figure 3). In this group, 26 (60%) symptomatic subjects were sensitized to milk, 28 (65%) patients were sensitized to egg. Sensitization to wheat was confirmed in 8 patients, to peanut, fish, and potato, in 1 patient, respectively. Sensitization to more than one food was detected in 20 (47%) patients (Figure 4).

 The sensitization level to food allergens demonstrated in the current study is lower than that in the other European birth cohorts. For example, 13% of Dutch infants are allergic to cow's milk as reported for the Dutch birth cohort KOALA [16], 11% of infants are allergic to eggs, and 0.5% to peanuts [9, 16], The prevalence of food allergy in infants varied from 9% as reported for the German LISA cohort [21] to 16% as reported for the MASS-90 cohort [6].

**Figure 5:**
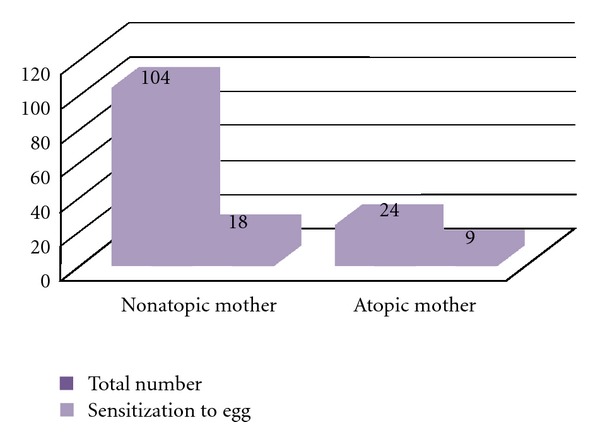
Relationship between maternal allergy and sensitization to egg of infants at the age of 12 months. Total numbers of infants and the numbers of infants sensitized to egg in each group are indicated.

The relationship between maternal diseases, the use of antibiotics and maternal diet during pregnancy, and sensitization of children to food allergens has been analysed in the group of 128 infants at the age of 12 months: 43 symptomatic and 85 control infants. There was no significant impact of maternal diseases and the use of antibiotics during pregnancy on early sensitization to food allergens (*P* > 0.05). Maternal avoidance of milk and egg products during pregnancy as well as consuming in elevated amounts of the respective product was not related to early sensitization to milk and egg allergens (*P* > 0.05). Parental allergic diseases had no significant impact on developing the sensitization to milk in children under 12 months of age (data not shown). However, infants of atopic mothers were significantly more often sensitized to egg (37.5%) as compared to the group of infants of nonatopic mothers (17.3%, *P* < 0.05), Figure 5.

 The sensitization to egg was studied in several previous cohorts [7, 18, 22]. It is supposed that the early sensitization to hen's egg might serve as a prognostic marker for later development of asthma and allergic rhinoconjunctivitis [3, 13, 16]. However, there are no direct evidences on the principal role of the egg sensitization in the development of astma. Therefore, our further attempts will be focussed for studying the relationship between maternal allergy and early sensitization to egg within the “Alergemol” cohort.

In summary, the frequency of food sensitization in early age determined in the Lithuanian “Alergemol” birth cohort differs from that reported in the other cohort studies. The differences in the prevalence of food allergy may be explained by different socioeconomic situations in the countries, national habits and foods. The followup of the Lithuanian birth cohort will provide new data on the allergy sensitization pattern in elder children and possible risk factors associated with later asthma development.

## 4. Conclusions

Investigation of early allergic sensitization in the Lithuanian birth cohort “Alergemol” demonstrated the increase of the incidence of food allergy during the first year of life. Sensitization to food allergens was determined in 1.3% of infants at 6 months of age and in 2.8% of infants at 12 months of age. The rate of food sensitization in early age was different from that reported for other European cohorts. Maternal diet, maternal diseases, the use of antibiotics, tobacco smoke or passive smoking during pregnancy had no significant impact on the early sensitization to food allergens. However, infants of atopic mothers were significantly more often sensitized to egg as compared to the infants of nonatopic mothers. Further studies on the food allergy sensitization in the “Alergemol” birth cohort will be directed in defining the possible risk factors associated with later asthma development.

## Figures and Tables

**Table 1:** Characteristics of the Lithuanian birth cohort “Alergemol” and the number of self-reported cases of allergy and food hypersensitivity of the family members.

	Cohort size: *n* = 1558
*Baby*	
Male gender (%)	51.4
Caesarean section (%)	15.7
*Mother*	
Age (years, mean ± SD)	28.3 ± 5.3
Allergy, self-reported (%)	5.9
Food hypersensitivity, self-reported (%)	5.1
*Father*	
Age (years, mean ± SD)	30.9 ± 6.2
Allergy, self reported (%)	2.8
Food hypersensitivity, self-reported (%)	1.0
*Family members*	
Atopy, self reported (%)	12.3
Food hypersensitivity, self-reported (%)	9.7

**Table 2:** Environmental exposures of mothers and infants in the Lithuanian birth cohort “Alergemol”.

	Cohort size: *n* = 1558
*Cigarette smoking*	
During pregnancy (%)	7.8
Passive exposure (%)	8.3
*Housing*	
Urban (%)	86.4
Rural, not farm (%)	9.5
Rural, farm (%)	4.1
*Pet ownership*	
Cats (%)	22.0
Dogs (%)	21.6
Other pets (%)	12.3
Farm animals (%)	2.7
*Flooring in baby's room*	
Carpet (%)	30.2
Wood laminate (%)	62.1
Linoleum tiles (%)	6.6
Ceramic tiles (%)	0.5
*Baby's mattress*	
Plastic cover (%)	9.6
Synthetic, for example, foam (%)	25.3
